# Implementation and Evaluation of the WADGPS System in the Taipei Flight Information Region

**DOI:** 10.3390/s100402995

**Published:** 2010-03-30

**Authors:** Shau-Shiun Jan, Shih-Chieh Lu

**Affiliations:** Institute of Civil Aviation, National Cheng Kung University, Tainan 70101, Taiwan; E-Mail: jazzisaac@gmail.com

**Keywords:** GPS, Wide Area Differential GPS (WADGPS), integrity, aircraft approach

## Abstract

This paper describes the implementation of the Wide Area Differential Global Positioning System (WADGPS) system in order to evaluate the operational performance of a satellite based aviation navigation system within Taipei Flight Information Region (FIR). The main objective of the WADGPS is to provide real time integrity information regarding the use of GPS for civil aviation applications. This paper uses the e-GPS observation stations operated by the Taiwan Ministry of Interior (MOI) as the WADGPS reference stations to collect the L1-L2 dual-frequency GPS measurements. A WADGPS master station is also implemented to process all GPS measurements sent from each reference station, and then generate the vector corrections. These vector corrections consist of the satellite ephemeris and clock errors, and a grid of ionospheric delays. The data stream also includes confidence bounds for the corrections and “Use/Do Not Use” messages to provide integrity. These messages are then passed to the WADGPS user through the Internet. This paper discusses the WADGPS system architecture and the system performance analysis. A five-day operation performance in Taipei Flight Information Region (FIR) is presented in this paper. The results show that the WADGPS can improve the accuracy performance of GPS positioning and fulfill the integrity performance required by Non-Precision Approach (NPA) defined by the International Civil Aviation Organization (ICAO).

## 1. Introduction

**Figure 1.**
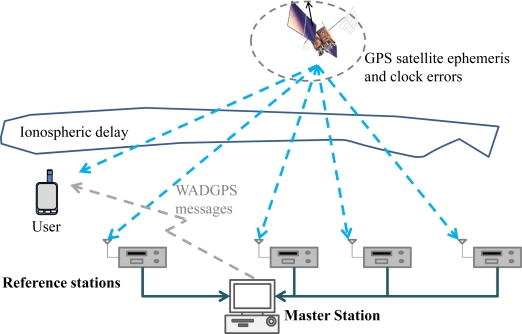
The Wide Area Differential GPS.

The Global Positioning System (GPS) provides positioning, navigation and timing services to around 400 million users in sea, air, terrestrial, and space applications [1]. In order to improve the performance of GPS-based navigation system providing support for the very demanding requirements of aircraft approach operations, various augmentation systems are being developed [2–4]. One such system is the Wide Area Differential GPS (WADGPS) system. The WADGPS system utilizes a geographically distributed network of reference receivers at precisely known locations throughout the service region, and these reference receivers, which are called Reference Stations (RSs), continuously monitor all GPS satellites and their propagation environments in real time. These RSs send raw GPS measurements back to the WADGPS Master Station (MS) where the WADGPS messages are generated. The WADGPS system provides both differential corrections to improve accuracy and associated confidence bounds to ensure integrity. The corrections will improve the accuracy of the system and more importantly, the integrity will open the door for widespread aviation use. The WADGPS concept is summarized in Figure 1. The Satellite Based Augmentation System (SBAS) is an extension of the WADGPS concept and there are several SBASs which are developing around world to enhance aviation navigation performance. For instance, the MTSAT-based Satellite Augmentation System (MSAS) is deployed in Japan, the European Space Agency (ESA) is working on the European Geostationary Navigation Overlay Service (EGNOS), and the Wide Area Augmentation System (WAAS) is under development in the United States [4]. Among them, WAAS began operation on July 10, 2003, MSAS was commissioned for aviation use on September 27, 2007, and EGNOS open service started on October 1, 2009. However, in Taipei Flight Information Region (FIR), there is no SBAS service available for aviation users. Therefore, the objective of this paper is to implement a WADGPS system to investigate the augmented GPS performance for civil aviation users within the Taipei FIR.

Similar to this work, in the early 1990s, the Federal Aviation Administration (FAA) implemented the National Satellite Test Bed (NSTB) as a WAAS prototype system to ensure the success of the WAAS. In 2006, the authors co-developed one of the RSs of the Asia-Pacific Economic Cooperation (APEC) Global Navigation Satellite System (GNSS) test bed which is a WADGPS-like system to conduct the preliminary analysis of the SBAS performance within Asia-Pacific [5]. With the experience of developing the WADGPS RS, this paper further implements the WADGPS master station to assess the performance of various architectures for the WADGPS. The e-GPS observation stations operated by the Taiwan Ministry of Interior (MOI) are used as the WADGPS RSs. Our focus will be one particular aircraft approach procedure known as Lateral NAVigation (LNAV), and the Required Navigation Performance (RNP) for LNAV is summarized in Table 1 [6,7]. The metrics used to quantify the performance will be the positioning accuracy and the service quality at a pre-defined and fixed level of integrity.

Accordingly, this paper is organized as follows: In Section 2, we define the metrics that are normally used to quantify the performance of GPS-based aircraft approach systems. In Section 3, we describe the details of the WADGPS architecture. Section 4 shows the main processes of the WADGPS master station. In Section 5, we first validate the implementation of the WADGPS using the U.S. NSTB data, and we then conduct several experiments to evaluate the LNAV performance of the WADGPS within Taipei FIR. Finally, Section 6 presents the summary and concluding remarks.

## 2. Performance Analysis of the WADGPS System

The Protection Level (PL) calculation [8] and Stanford Chart [9,10] are used as the metrics to evaluate the performance of a GPS-based aircraft approach and landing system. The performance criterion chosen for this paper is the comparison of the LNAV availability. The PL is the computation of the confidence bounds on the post-correction position errors, and the PL calculation is defined in the Minimum Operational Performance Standards for GPS/WAAS Airborne Equipment (WAAS MOPS, RTCA DO-229D) [8]. The ionospheric delay error and satellite ephemeris and clock errors will be corrected according to the WAAS MOPS, and then the local errors such as the tropospheric delay error and user receiver noise and multipath errors will be removed by a standard model [11]. The corrected range measurements are used to compute the GPS position and receiver clock errors using weighted least squares as follows:

(1) $$\hat{x}={\left(G^{T}\mathrm{WG}\right)}^{-1}G^{T}\mathrm{Wy}$$

where:

*x̂* is the position and clock errors,
*G* is the observation matrix,
*W* is the weighting matrix for the measurement, and
*y* is the corrected range residual vector.

The weighting matrix, *W*, is a diagonal matrix and the inverse of the *i^th^* diagonal element is given by the variance for the corresponding satellite, as depicted in Equation (2).

(2) $$W^{-1}=\left[\begin{array}{cccc} \sigma_{1}^{2} & 0 & \cdots & 0\\ 0 & \sigma_{2}^{2} & \cdots & 0\\ \vdots & \vdots & \ddots & \cdots \\ 0 & 0 & \cdots & \sigma_{i}^{2} \end{array}\right]$$

where, $\sigma_{i}^{2}$ is calculated in Equation (3).

(3) $$\sigma_{i}^{2}=\sigma_{i,\mathrm{flt}}^{2}+\sigma_{i,\mathrm{UIRE}}^{2}+\sigma_{i,\mathrm{air}}^{2}+\sigma_{i,\mathrm{tropo}}^{2}$$

where:

$\sigma_{i,\mathrm{flt}}^{2}$ is the fast and long-term degradation confidence, which is the confidence bound on satellite clock and ephemeris corrections [8,12],
$\sigma_{i,\mathrm{UIRE}}^{2}$ is the user ionospheric range error confidence, which is the confidence bound on ionospheric delay corrections [8,11],
$\sigma_{i,\mathrm{air}}^{2}$ is the airborne receiver error confidence, which is the confidence bound on aircraft user receiver error [8], and
$\sigma_{i,\mathrm{tropo}}^{2}$ is the tropospheric error confidence, which is the confidence bound on residual tropospheric error [8].

The position error is proportional to the measurement errors and the satellite geometry through the matrix (*G*^*T*^*WG*)^−l^. This matrix is composed of the variance from each direction as indicated in Equation (4). The variance of the position estimate is shown in Equation (4) as well:

(4) $${\left(G^{T}\mathrm{WG}\right)}^{-1}=\left[\begin{array}{cccc} d_{\mathrm{east}}^{2} & d_{\mathrm{EN}} & d_{\mathrm{EU}} & d_{\mathrm{ET}}\\ d_{\mathrm{EN}} & d_{\mathrm{north}}^{2} & d_{\mathrm{NU}} & d_{\mathrm{NT}}\\ d_{\mathrm{EU}} & d_{\mathrm{NU}} & d_{U}^{2} & d_{\mathrm{UT}}\\ d_{\mathrm{ET}} & d_{\mathrm{NT}} & d_{\mathrm{UT}} & d_{T}^{2} \end{array}\right]$$

where:

$d_{\mathrm{east}}^{2}$ is variance in the east direction,
$d_{\mathrm{north}}^{2}$ is variance in the north direction,
$d_{U}^{2}$ is variance in the up direction,
$d_{T}^{2}$ is variance in time, and
$d_{\left(i)(j\right)}^{2}$ is covariance in the (*i*) and (*j*) directions, where *E* is east, *N* is north, *U* is up, and *T* is time.

The Horizontal Protection Level (HPL) is:

(5) $$\mathrm{HPL}=K_{H}\cdot \sqrt{\frac{d_{\mathrm{east}}^{2}+d_{\mathrm{north}}^{2}}{2}+\sqrt{{\left(\frac{d_{\mathrm{east}}^{2}-d_{\mathrm{north}}^{2}}{2}\right)}^{2}+}d_{\mathrm{EN}}^{2}}$$

where, the *K_H_* equals to 6.18 for Non-Precision Approach (NPA). This is a standard deviation multiplier of the horizontal position error. Through the zero mean Gaussian [13], the multiplier ensures that the horizontal position error exceeds the HPL at most one time in ten million (10^−7^), the tolerable probability of Hazardously Misleading Information (HMI). The protection level calculation is specified in the WAAS MOPS Appendices A and J [8]. The HPL is very important and will be used to determine the performance of WADGPS system in this paper.

**Figure 2.**
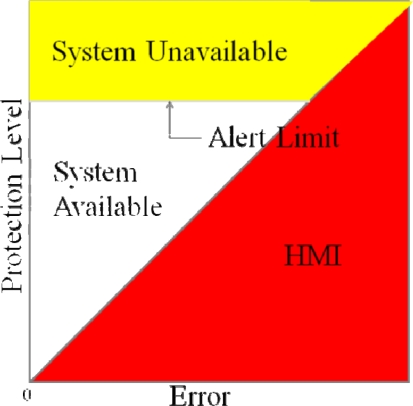
The Stanford Chart.

On the other hand, this paper uses a triangular chart as our WADGPS system performance indicator, called the Stanford Chart [9]. The Stanford Chart helps us visualize the performance of the corrections and error bounds of a GPS integrity messaging system. The chart helps evaluate availability, accuracy, and integrity. The performance is evaluated in the user position domain in a two-dimensional space. The horizontal axis represents the true position error magnitude, and the vertical axis represents the estimated protection level described above. One Stanford Chart example is shown in Figure 2. There are three major regions: *System Available*, *System Unavailable*, and *Hazardously Misleading information (HMI)*. If the estimated protection level is smaller than the alert limit of a designed operation mode, the service will be *available.* If the estimated protection level is larger than the alert limit of a designed operation mode, the service will be *unavailable*. If the true error is larger than the estimated protection level, it is *hazardously misleading information*, because the protection level is meant to bound the true error and users have no knowledge of the excessive true error. This situation should occur with a probability of less than one in ten million operations or 10^−7^ and should be avoided. As a result, this Stanford Chart can intuitively present system performance in terms of accuracy, availability and integrity. The availability of the system can be determined by examining the percentage of points that lie within the service available region [14].

## 3. WADGPS Architecture

**Figure 3.**
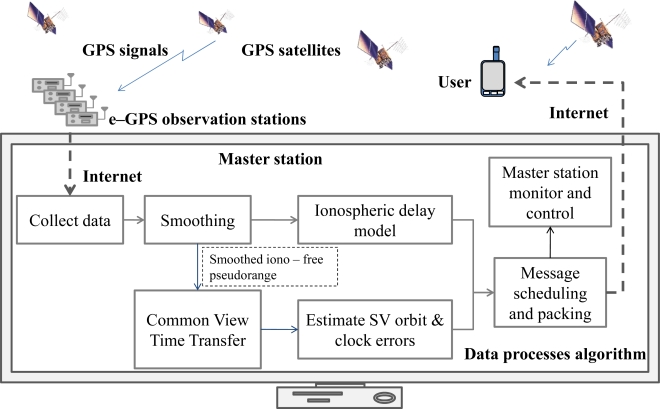
The WADGPS architecture.

The WADGPS is a network composed of several Reference Stations (RSs) and a Master Station (MS). The RSs are distributed geographically at the precisely known locations that receive GPS L1-L2 dual-frequency signals and archive the raw observations from the monitored GPS satellites. The GPS L1-L2 dual-frequency measurements collected at each RS are sent to the MS. The MS data collector receives GPS raw measurements from each RS and updates the previous measurements in real time. Moreover, the statuses of GPS signals for all monitored satellites are checked including the rationalities of the code and carrier phase measurement at L1 and L2 frequencies, Signal-to-Noise Ratios (SNR), and Doppler frequency. The raw GPS observations are subsequently processed to reduce local errors by the carrier smoothing method [11]. The WADGPS MS then uses these smoothed measurements to generate vector corrections for the ionospheric delay, and the satellites ephemeris and clock errors [3]. In addition to these vector corrections, the messages generated by the MS also include the confidence bounds of these corrections. These messages are packed into the SBAS message format [8] and then transmitted to users *via* Internet according to the appropriate scheduling time. The WADGPS MS implemented in this paper includes a monitor and control graphic user interface (GUI) to show the real time statuses of all RSs and MS processes. Figure 3 shows a diagram that summarizes the overall WADGPS system architecture and data processes.

## 4. The WADGPS Master Station Processes

The WADGPS master station (MS) receives and processes the measurements from all WADGPS reference stations (RSs). The data collected from each RS is calibrated and used to generate the differential corrections to ionosphere and satellite errors. There are two main correction generation modules: one is for the ionosphere and the other is for satellite errors. WADGPS provides the user with the differential corrections and two system accuracy metrics, namely, the user differential range error (UDRE) and the grid ionospheric vertical error (GIVE) [8]. This section begins with the introduction of GPS observations, and then presents the dual-frequency carrier smoothing process in Section 4.2. The estimation of ionospheric delay and the calculation of GIVE will be detailed in Section 4.3 and the estimation of satellite errors and the calculation of UDRE will be described in Section 4.4.

### 4.1. GPS Observations Modeling

Each RS uses a dual-frequency receiver to receive code and carrier phase observations at the L1 and L2 frequencies. These raw observations are sent to the WADGPS master station to process the corrections for common errors and the corresponding confidences [3,4]. The common errors include the ionospheric delay and satellites ephemeris and clock errors. The observations are expressed as follows [1,15]:

(6) $${\mathrm{PR}}_{L1}=\rho_{i}^{j}+b_{i}-B^{j}+I_{i}^{j}+T_{i}^{j}+\nu_{i,L1}^{j}$$

(7) $${\mathrm{PR}}_{L2}=\rho_{i}^{j}+b_{i}-B^{j}+\gamma I_{i}^{j}+T_{i}^{j}+\nu_{i,L2}^{j}$$

(8) $$\phi_{L1}=\rho_{i}^{j}+b_{i}-B^{j}-I_{i}^{j}+T_{i}^{j}+N_{L1}\lambda_{L1}+e_{i,L1}^{j}$$

(9) $$\phi_{L2}=\rho_{i}^{j}+b_{i}-B^{j}-\gamma I_{i}^{j}+T_{i}^{j}+N_{L2}\lambda_{L2}+e_{i,L2}^{j}$$

(10) $$\gamma ={\left(\frac{L_{1}}{L_{2}}\right)}^{2}={\left(\frac{1575.42}{1227.6}\right)}^{2}=1.647$$

where:

*PR* is the pseudorange and the subscripts *L*1 and *L2* indicates L1 and L2 frequencies, respectively,
$\rho_{i}^{j}$ is the geometric range from satellite *j* to user *i*,
*ϕ* is the carrier phase and the subscripts *L*1 and *L2* indicates L1 and L2 frequencies, respectively,
*b* is the receiver clock bias,
*B* is the satellite clock error,
*Nλ* is the integer ambiguities and the subscripts *L*1 and *L2* indicates L1 and L2 frequencies, respectively,
*I* is the ionospheric delay at L1 frequency,
*T* is the tropospheric delay,
*v* is the pseudorange measurements noise and the subscripts *L*1 and *L2* indicates L1 and L2 frequencies, respectively, and
*e* is the carrier phase measurements noise and the subscripts *L*1 and *L2* indicates L1 and L2 frequencies, respectively.

The differences in these observation equations are the ionospheric delays. The pseudorange (code phase) measurement is delayed and the carrier phase is advanced, and this is the reason of the sign difference of *I* in Equations (6–7) and Equations (8–9). This delay is inversely proportional to the signal frequency [1]. In Equations (7) and (9), *γ* equals 1.647. Thus, the ionospheric delay on L2 is 1.647 times larger than that on L1. Additionally, the carrier phase observations also suffer from integer ambiguity (*Nλ*) [1].

### 4.2. Dual-Frequency Carrier Smoothing of Pseudorange and Ionospheric Delays

To mitigate the measurements noise and multipath effects, a dual-frequency carrier smoothing filter is used after raw GPS observations collecting from each RS [11]. Because the measurement noise of the carrier phase observations are much smaller than that of the pseudorange measurements, the pseudorange and carrier phase are combined to reduce the measurement noise [1]. The smoothing filter is implemented using three ionospheric measurements from the dual-frequency observables, and the filter design is detailed in [11]. The ionospheric delay measurements could be derived by the linear combination of GPS L1 and L2 pseudorange and carrier phase observables [11]:

(11) $$I_{L1,\mathrm{PR}}\equiv \frac{{\mathrm{PR}}_{L2}-{\mathrm{PR}}_{L1}}{\gamma -1}=I_{L1}+v_{\mathrm{PR}}$$

(12) $$I_{L1,\phi }\equiv \frac{\phi_{L1}-\phi_{L2}}{\gamma -1}=I_{L1}+\mathrm{Amb}+v_{\phi }$$

(13) $$I_{L1,L1}\equiv \frac{{\mathrm{PR}}_{L1}-\phi_{L1}}{2}=I_{L1}-\frac{N_{L1}\lambda_{L1}}{2}+v_{L1}$$

where:

*I*_*L*1_ is ionospheric delay at the L1 frequency, the extra subscripts present the observations used in the combination,
*Amb* is the combination of ambiguities from the L1 and L2 carrier phases, and the magnitude of noises are v_*PR*_ > v_*L*1_ > v_*ϕ*_[1].

**Figure 4.**
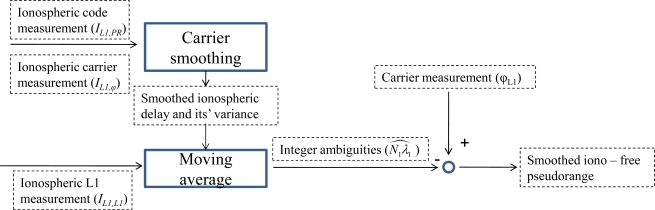
The dual-frequency smoothing of ionospheric delay and pseudorange.

The dual-frequency carrier smoothing filter is depicted in Figure 4. The filter estimated the smoothed ionospheric delay, *Î*_*smth*_, and smoothed ionosphere-free pseudorange, *PR*_*L*1_. The first step in the filter is to generate *Î*_*smth*_ and its confidence by smoothing the *I*_*L*1,*PR*_ with the low noise *I*_*L*1,*ϕ*_. Then combining the *Î*_*smth*_ and Equation (14) to estimate the constant *N*_*L*1_*λ*_*L*1_ by moving average. If the cycle slip is not present, the N_*L*1_λ_*L*1_ is constant. Finally, substituting *Î*_*smth*_ and N_*L*1_*λ*_*L*1_ into the L1 carrier phase, *ϕ*_*L*1_ (*i.e.*, Equation (8)), the ionosphere-free pseudorange, *PR*_*L*1_, is obtained [11].

### 4.3. Ionospheric Corrections

The major functions of the WADGPS master station (MS) are the ionospheric corrections model and the satellites ephemeris and clock errors estimation algorithms. After the dual-frequency carrier smoothing filter outputs the smoothed ionospheric delay, the MS then converts all ionospheric slant delays to the vertical delays at the Ionosphere Pierce Points (IPPs) by the Obliquity Factor (*OF*) [11]. By doing so, the ionospheric measurement is independent of the elevation angle, and it will be more convenient to use. The location of IPP is defined as the intersection of the line segment from the RS to the satellite and an ellipsoid with constant high above 350 km from earth’s surface [16]. The next step is to create a vertical ionospheric delay model from the IPP measurements to estimate the ionospheric vertical delay at the Ionosphere Grid Points (IGP), *Î*_*G*_. The Grid Ionospheric Vertical Error (*GIVE*) is provided for each IGP which is a confidence bound of the corrected ionospheric delay residual at the IGP. The following Equations (15) and (16) estimate the ionospheric vertical delay at the IGP and *GIVE* by the weighted least-squared algorithm, and the derivations of these equations and *OF* are detailed in [11]:

(14) $${\hat{I}}_{G}=I_{\mathrm{Klobuchar},G}\cdot \sum_{i=1}^{k}\left(\frac{I_{\mathrm{measure},i}}{I_{\mathrm{Klobuchar},i}}\cdot \omega_{i}\right)/\sum_{i=1}^{k}\omega_{i}$$

(15) $$\mathrm{GIVE}=3.29/\sum_{i=1}^{k}\omega_{i}$$

where:

*ω*_*i*_ is the weight of the *i*^*th*^ IPP measurement,
*I*_*Klobuchar,G*_ is the vertical ionospheric delay at the grid point using the Klobuchar model parameters [1],
*I*_*Measure,i*_ is the vertical ionospheric delay measurement at the pierce point, and
*I*_*Klobuchar,i*_ is the vertical ionospheric delay at the pierce point using the Klobuchar model parameters.

The weight is calculated by the inverse of the vertical delay measurements variance according to the correlation distance between the grid point and the IPP as shows in Equation (17) [11].

(16) $$\omega_{i}=\frac{\sigma_{i}}{\Delta }$$

where:

*σ*_*i*_ is *i*^*th*^ vertical ionospheric delay measurements variance [11], and
Δ is a function of the correlation distance of the ionosphere [11].

**Figure 5.**
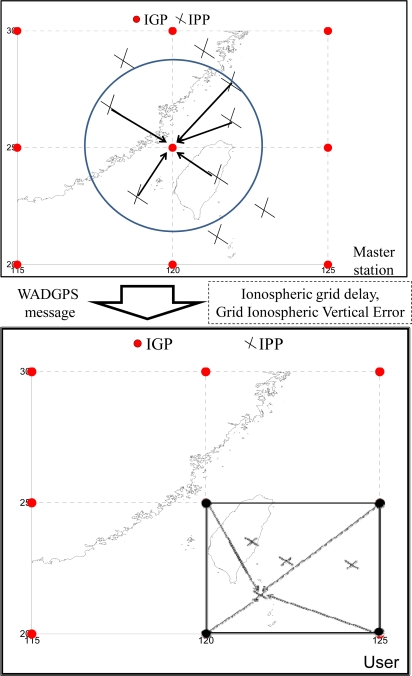
The WADGPS ionospheric vertical delay grid model flow chart.

Specifically, this model scales the measurements using the Klobuchar model to transport the measurement from the IPP location to the location of the desired grid point through the relationship of latitude and longitude dependence provided by the Klobuchar model [16]. The generation process of this grid model is illustrated in the upper plot of Figure 5.

The bottom plot of Figure 5 describes the ionospheric correction algorithm for WADGPS user receiver which uses the nearest IGPs around the IPP to estimate the vertical ionospheric delay at a specific IPP by the interpolation algorithm. The interpolation algorithm is expressed as:

(17) $$I_{\mathrm{IPP},V,i}^{\mathrm{Esti}}=\sum_{i=1}^{4}W_{i}\left(x_{\mathrm{IPP},i},y_{\mathrm{IPP},i}\right)\cdot I_{\mathrm{IGP},V,i}$$

(18) $${\mathrm{UIVE}}_{\mathrm{IPP},i}=\sum_{i=1}^{4}W_{i}\left(x_{\mathrm{IPP},i},y_{\mathrm{IPP},i}\right)\cdot {\mathrm{GIVE}}_{i}$$

where:

$I_{\mathrm{IPP},V,i}^{\mathrm{Esti}}$ is the vertical ionospheric delay at the *i*^*th*^ IPP, estimated with the broadcasted ionospheric corrections,
*W*_*i*_(*x*_*IPP,i*_,*y*_*IPP,i*_) is the weighting factor of the *i*^*th*^ IPP whose location is (*x*_*IPP,i*_,*y*_*IPP*_,_*i*_) [8],
*I*_*IGP,V,i*_ is the broadcast vertical ionospheric delay at *i*^*th*^ IGP,
*UIVE*_*IPP,i*_ is the user ionospheric vertical error (UIVE) which is a 99.9% confidence (error bound) on the post-correction ionospheric vertical delay residual [8], and
*GIVE*_*i*_ is a confidence bound of the corrected ionospheric delay residual at the *i*^*th*^ IGP.

**Figure 6.**
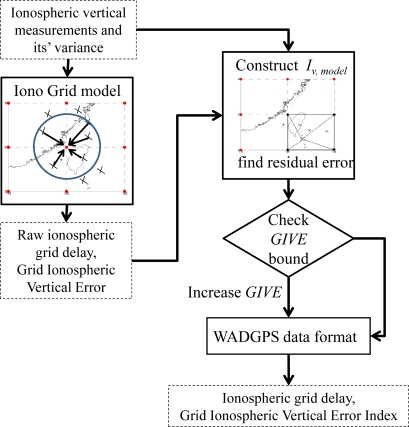
The feedback algorithm for GIVE.

The MS generates the grid model and its confidence with feedback information to ensure that GIVE covers 99.9% of the corrected ionospheric residuals statistically. Therefore, the MS uses the grid model to estimate the vertical ionospheric delays of the RSs and their confidences (UIVE). Then, the master station can determine if the UIVE bounds the difference of ionospheric delays from the grid model (based on above user algorithms, Equations (17)) and that from the RSs’ own dual-frequency measurements. If not, the MS must increases the GIVEs of the four grid points surrounding the IPP measurement. After checking all IPPs from the entire network, the GIVEs are guaranteed to cover 99.9% of the corrected ionospheric residuals statistically [11]. Figure 6 summarizes this process.

### 4.4. Satellite Ephemeris and Clock Corrections

**Figure 7.**
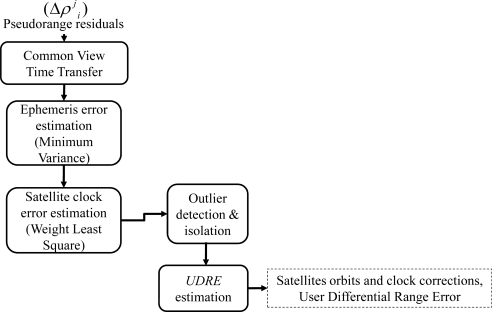
Ephemeris and clock errors estimation flow chart.

This section describes the MS procedures for satellite ephemeris and clock errors estimations. Figure 7 depicts the flow chart regarding the calculations of the satellites ephemeris and clock errors including the Common View Time Transfer (CVTT), ephemeris error estimation, satellite clock error estimation, and the User Differential Range Error (UDRE) estimation [4,12].

**Figure 8.**
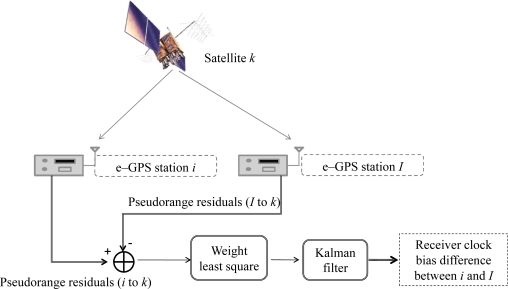
The common view time transfer flow chart.

The CVTT filter synchronizes the measurements with a common reference time and decouples the measurements sequentially for each satellite to eliminate the receiver clock bias. To find the difference of the clock biases between two RSs, CVTT filter obtains the synchronized pseudorange residuals from the first difference between the pseudorange residuals of two stations as shown in Equation (19), and the CVTT implementation is illustrated in Figure 8 [12]:

(19) $$\Delta_{i,I}^{j}=\Delta \rho_{i}^{j}-\Delta \rho_{I}^{j}=\Delta R^{j}\cdot \left(l_{i}^{j}-l_{I}^{j}\right)+\Delta b_{i,I}+\nu_{i,I}^{j}$$

where:

Δ*R*^*j*^ is the ephemeris error,
$l_{i}^{j}$ is the unit line of sight vector from the *i* RS to the *j* satellite,
$l_{I}^{j}$ is the unit line of sight vector from the *I* RS to the *j* satellites,
$\left(l_{i}^{j}-l_{I}^{j}\right)$ is the line of sight difference,
Δ*b*_*i*_,_*I*_ is the clock difference, and
$\nu_{i,I}^{j}$ is the measurement noise.

Then, the clock bias difference, Δ*b̂*_*i,I*_, is described in Equation (20):

(20) $$\Delta {\hat{b}}_{i,I}=\frac{1}{k}\sum_{j=1}^{k}\Delta_{i,I}^{j}$$

where *k* is the number of satellites in the common view of both RSs.

Through the CVTT module, the pseudorange residuals from all RSs are synchronized based on a common clock, and the pseudorange residuals consist of satellite ephemeris and clock errors. The corrections to the ephemeris error and the clock error have to be sent frequently, and they occupy lots of bandwidth. To reduce the bandwidth, separating the satellite clock error term is necessary. Therefore, the single difference is used to remove the satellite clock error term as shown in the following equation:

(21) $$\Delta {\tilde{\rho }}_{i}^{j}-\Delta {\tilde{\rho }}_{m}^{j}=\Delta R^{j}\cdot \left(l_{i}^{j}-l_{m}^{j}\right)+\varepsilon_{i}^{j}$$

where:

Δ*R*^*j*^ is ephemeris error which is this process solving for, and
the subscript “*m*” denotes the key RS which has the smallest variance [12].

Then, the Equation (21) is re-written in matrix forms as follows:

(22) $$z=H_{e}\cdot \Delta R^{j}+\nu$$

where:

$$z=\left[\begin{array}{c} \Delta {\tilde{\rho }}_{1}^{j}-\Delta {\tilde{\rho }}_{m}^{j}\\ \vdots \\ \Delta {\tilde{\rho }}_{N-1}^{j}-\Delta {\tilde{\rho }}_{m}^{j} \end{array}\right],H_{e}=\left[\begin{array}{c} l_{1}^{j}-l_{m}^{j}\\ \vdots \\ l_{N-1}^{j}-l_{m}^{j} \end{array}\right]$$

Δ*R*^*j*^ is the ephemeris error which will be denoted as *x* in following discussions,
*N* is number of synchronized RSs, and
*v* is measurement noise with zero mean and variance of *W*.

As indicated in Equation (23), the satellite position errors are estimated by the minimum variance estimator [4]:

(23) $${\hat{x}}_{MV}={\left(\Lambda^{-1}+H^{T}W^{-1}H\right)}^{-1}H^{T}W^{-1}z$$

where:

$$\Lambda =E\left[xx^{T}\right],W=\text{cov}(\nu )$$

After estimating the ephemeris error by the minimum variance method, the clock error measurements for all satellites can be derived from the synchronized pseudorange residuals. Equation (24) shows the clock error measurements for the *j*^*th*^ satellite from the *i*^*th*^ RS [12]:

(24) $$z_{c,i}^{j}=\Delta {\hat{R}}^{j}\cdot l_{i}^{j}-\Delta {\tilde{\rho }}_{i}^{j}=\Delta B^{j}+n_{i}^{j}$$

Then, the Equation (24) is re-written in matrix forms as follows:

(25) $$z_{c}=H_{c}\Delta B^{j}+n_{c}$$

where:

the subscript *c* denotes clock,
*H*_*c*_ is a column vector with all 1’s,
*n*_*c*_ is the measurement noise with covariance matrix *W*_*c*_.

In Equation (26), a weighted least-square method is used to derive the satellite clock error [12].

(26) $$\Delta {\hat{B}}_{\mathrm{WLS}}^{j}={\left({H^{T}}_{c}{W^{-1}}_{c}H_{c}\right)}^{-1}{H^{T}}_{c}{W^{-1}}_{c}z_{c}$$

Finally, to bound and indicate the uncertainty of the satellite ephemeris and clock corrected pseudorange, UDRE is calculated for each visible satellite as in Equation (27) [17]:

(27) $$P_{\mathrm{UDRE}}=R+H\hat{P}H^{T}$$

where:

*R* is the measurement covariance matrix of the synchronized pseudorange residuals,
*P̂* is the covariance of the estimated ephemeris and clock errors, and
*H* is the design matrix composed by unit length line of sight vectors and satellites clock term, and the line of sight vectors cover all users inside the reference network [12].

The UDRE value is calculated in Equation (29):

(28) $$\sigma_{\mathrm{UDRE}}^{2}={\left(\sum_{i=1}^{i=m}\frac{1}{P_{\mathrm{UDRE},\mathrm{ii}}}\right)}^{-1}$$

(29) $$\mathrm{UDRE}=3.29\times \sigma_{\mathrm{UDRE}}^{2}$$

where *P*_*UDRE,ii*_ is the *i*^*th*^ diagonal element of the *P*_*UDRE*_.

When the WADGPS users receive the satellite ephemeris and clock corrections, the corrections need to be converted to the pseudorange domain. Equation (30) shows the pseudorange correction error for satellite *i* which is corrected by satellite ephemeris and clock errors, and this pseudorange correction error has to be bounded by the combined UDRE and pseudorange sigma values [12]:

(30) $$\rho_{\mathrm{corrected}}^{i}={\mathrm{PR}}^{i}-\Delta {\hat{R}}^{i}\cdot l^{i}+\Delta {\hat{B}}^{i}$$

where:

*PR*^*i*^ is pseudorange from the *i*^*th*^ visible satellite,
Δ*R*^*i*^ is satellite ephemeris corrections,
*l*^*i*^ is line of sight vector from the user to the satellite, and
Δ*B̂*^*i*^ is clock corrections.

## 5. Experiments and Performance Evaluation

To implement the WADGPS system in Taipei flight information region (FIR), the stable RSs collection of dual-frequency GPS observations are essential. This paper uses the e-GPS observation stations in Taiwan as the WADGPS RSs, and the e-GPS observation stations are operated by Taiwan Ministry of Interior. The WADGPS RSs send four types of raw dual-frequency GPS observations to the WADGPS MS including:

■ Range data: it is composed of L1-L2 dual-frequency pseudorange, carrier phase, Doppler frequency, and signal to noise ratio of each satellite in view by the network [1]. The data update rate is 1 Hz.
■ Ephemeris data [15]: it includes GPS orbit parameters and satellite clock correction coefficients of each satellite in view by the network. It is updated every 50 seconds.
■ Almanac data [15]: it consists of the simplified GPS orbit parameters. It is updated every 500 seconds.
■ The Klobuchar model coefficients [15]: it provides the common ionospheric model for single frequency users. It is updated every 500 seconds

### 5.1. WADGPS Implementation Procedures

**Figure 9.**
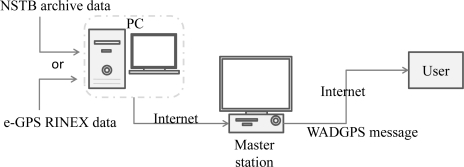
Experiment setup.

The WADGPS system implemented in this paper is based on that of the NSTB which is operated by FAA in the United States. Therefore, the NSTB archive data are used to verify the WADGPS performance. On the other hand, the GPS receivers used in the e-GPS observation stations might be different, for ease of data processing, the common GPS observation data format, the Receiver INdependent EXchange format (RINEX), is adopted for this work. Before the WADGS MS can use the observations to generate the WADGPS messages, the RINEX data needs to be decoded and organized in a proper format. Figure 9 shows the experiment setup. A computer is used to collect the RINEX data and NSTB archive data for data pre-process which transforms them into the WADGPS data format. This computer then sends the GPS observations to the WADGPS MS *via* the Internet to execute the WADGPS MS algorithms to generate the corresponding WADGPS messages. Finally, the WADGPS messages are sent to the WADGPS users *via* the Internet.

**Figure 10.**
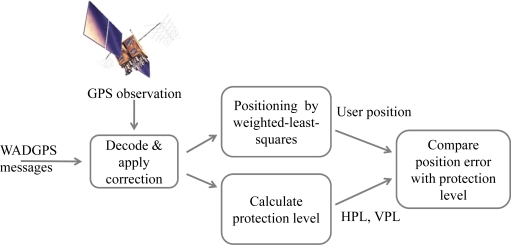
The procedures of the WADGPS user software.

**Figure 11.**
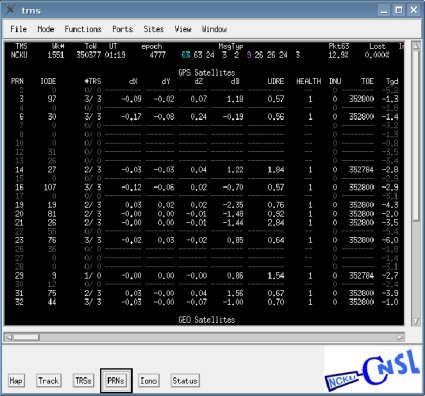
The WADGPS satellites corrections status window.

**Figure 12.**
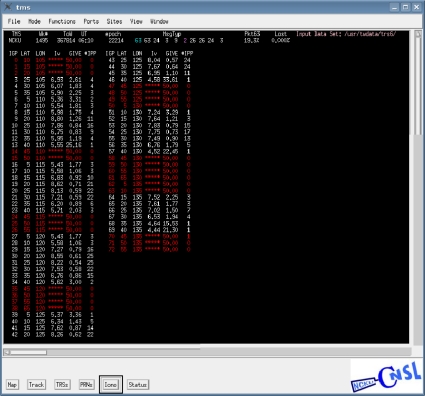
The WADGPS ionospheric grid corrections status window.

**Figure 13.**
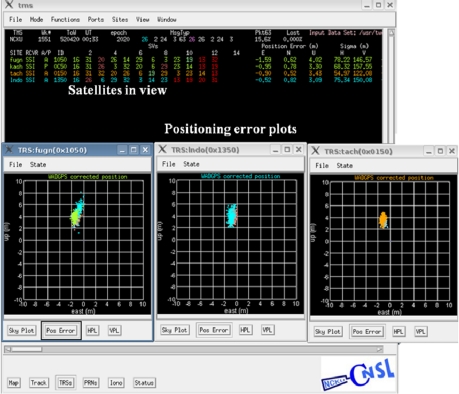
The WADGPS reference stations status window.

To evaluate the performance of the WADGPS developed in this work, a WADGPS user monitor is developed based on WAAS MOPS [8] and its flow chart is depicted in Figure 10. The operating system (OS) of the WADGPS user monitor is FreeBSD [18] and the process is developed using C language and Open Motif [19]. After receiving and decoding the WADGPS messages, the WADGPS user applies the vector corrections to the GPS measurements according to WAAS MOPS. In addition, the protection level (PL) is calculated based on the received integrity messages [8]. The Horizontal Protection Level (HPL) calculation is also defined in WAAS MOPS [8]. For the convenience of monitoring the WADGPS MS processes, this work also develops a Graphic User Interface (GUI) to show the WADGPS MS status. Figures 11 to 13 depict the master station monitor and control GUI. In Figure 11, the first row describes the GPS time and the message types generated by the master station. The GPS satellites corrections status window also includes the satellite position error corrections in the ECEF coordinate, satellite clock error corrections, the UDRE and the health flag for each satellite. For the ionospheric grid corrections, Figure 12 shows the ionospheric vertical delays and their GIVE values at the grid points. Figure 13 exhibits the reference stations status which includes their positioning results and the satellites in view. The other status windows include the reference stations distribution map, the ground tracks of the satellites in view, and the WADGPS system status.

### 5.2. WADGPS Performance Analysis

**Figure 14.**
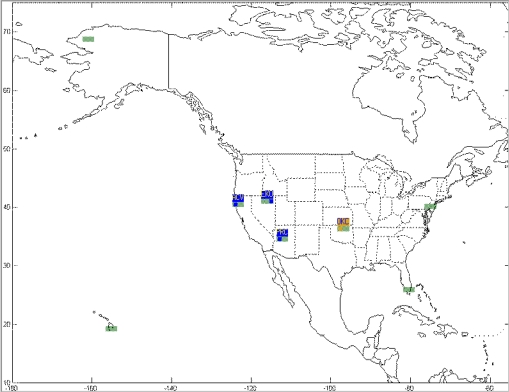
The NSTB reference stations distribution.

**Figure 15.**
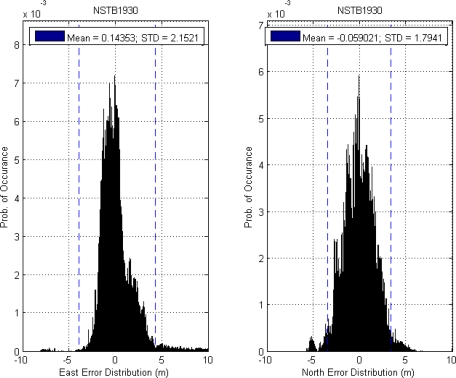
(a) The positioning error distribution and 95% error bound in east direction using NSTB data. (b) The positioning error distribution and 95% error bound in north direction using NSTB data.

**Figure 16.**
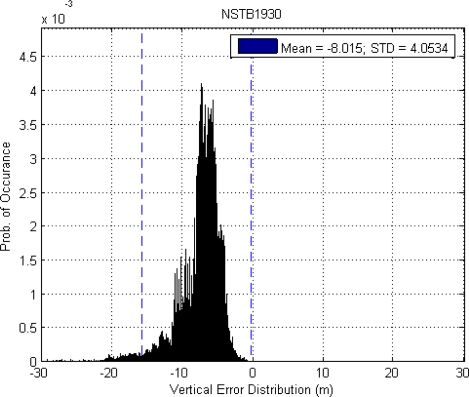
The vertical positioning error and 95% error bound using NSTB data.

**Figure 17.**
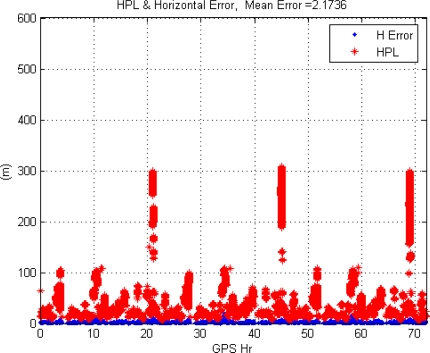
The HPL and horizontal positioning error (NSTB).

**Figure 18.**
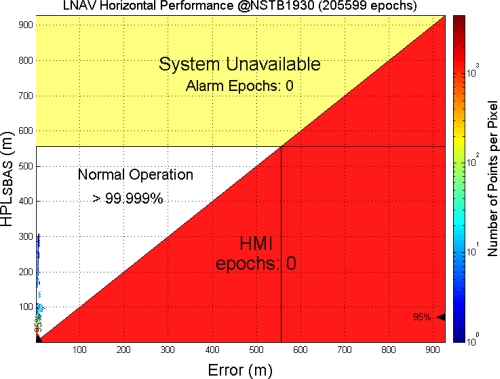
The LNAV (NPA) performance of the implemented WADGPS with NSTB data.

**Figure 19.**
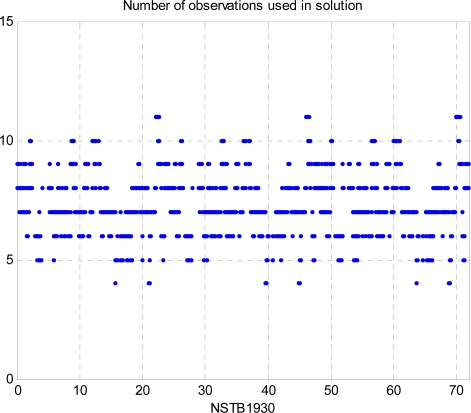
The number of satellites used in positioning solutions (NSTB).

This paper first used four NSTB reference stations to validate the implementation of the WADGPS system, and three of them are used as the WADGPS RSs and one acts as the WADGPS user. The RSs distribution is shown in Figure 14. The blue RSs are used as the WADGPS RSs, and the yellow RS is used as the WADGPS user. Three-day data is used in this process and they are dated from 2008/12/13 to 2008/12/15. Because the locations of these NSTB RSs are precisely known, we could use them to perform the positioning performance analysis. Figure 15 shows the positioning error distributions in both east and north directions when the WADGPS user applies the WADGPS messages generated by the implemented WADGPS MS with three NSTB RSs. The vertical positioning error distribution is shown in Figure 16. In these figures, the blue dashed line indicates the 95% error bounds on the positioning errors. To show the benefits of using the WADGPS corrections, comparisons of two positioning methods are summarized in Tables 2 and 3. The GPS positioning performance with the WADGPS corrections outperforms the stand alone GPS positioning performance. As a result, the positioning accuracy is improved by the implementation of WADGPS. Furthermore, this paper uses the same three-day data to verify the integrity of the WADGPS MS algorithms, and Figure 17 shows that the HPL values successfully bound the horizontal positioning errors. Figure 18 uses Stanford Chart to validate the LNAV performance of this WADGPS system. As shown in this figure, this WADGPS implementation could provide the LNAV service with no HMI for entire three days period (*i.e.,* availability > 99.999%). The number of satellites used in the solutions is shown in Figure 19. Thus, the WADGPS system implemented in this work is validated.

**Figure 20.**
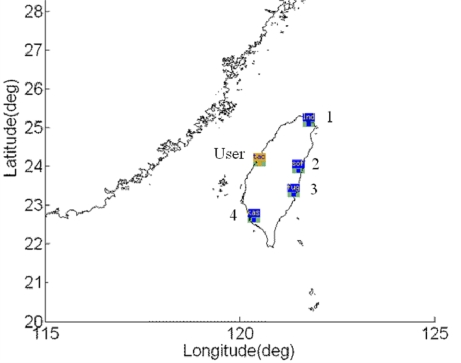
The e-GPS observation stations distribution map.

Next, this work uses the e-GPS observation stations operated by Taiwan MOI to evaluate the LNAV performance of the WADGPS implementation. The e-GPS observation stations distribution is shown in Figure 20, and their locations are listed in Table 4. In order to evaluate the performance change due to the number of RSs, this paper uses two kinds of WADGPS RSs constellations in Taipei FIR. One uses three RSs (*i.e.,* RS 1, RS 2 and RS 3) and the other uses four RSs (*i.e.,* RSs 1–4). The RS 5 is used as the WADGPS user in the experiments. Five-day data is used in the experiments and they are dated from 2009/10/01 to 2009/10/05.

**Figure 21.**
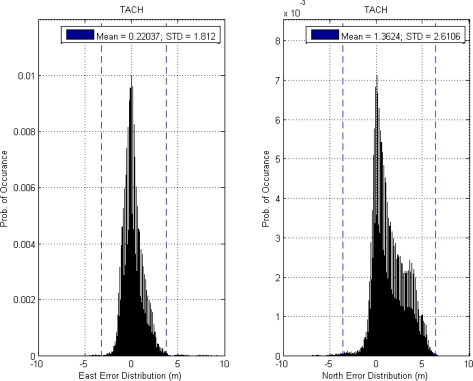
(a) The positioning error distribution and 95% error bound in east direction in Taipei FIR (3 RSs). (b) The positioning error distribution and 95% error bound in north direction in Taipei FIR (3 RSs).

**Figure 22.**
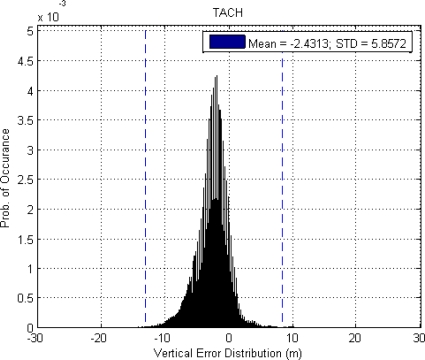
Te vertical positioning error and 95% error bound in Taipei FIR (3 RSs).

**Figure 23.**
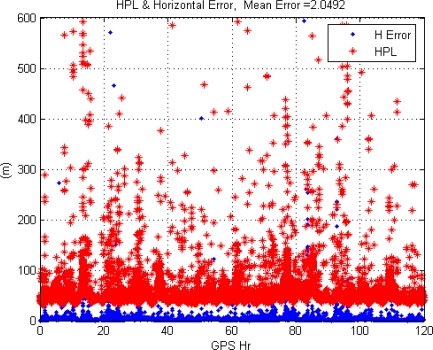
The HPL and horizontal position error in Taipei FIR (3 RSs).

**Figure 24.**
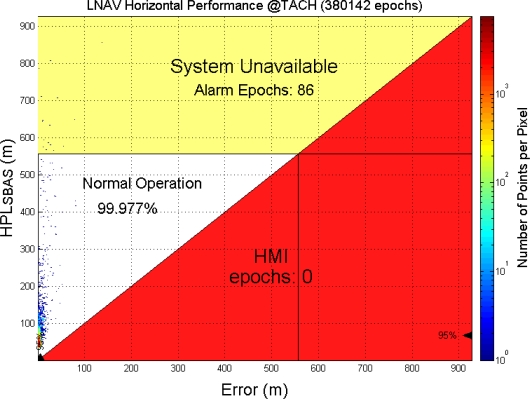
The LNAV (NPA) performance of the developed WADGPS with 3 RSs in Taipei FIR.

**Figure 25.**
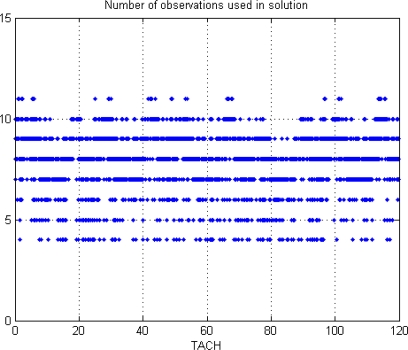
The number of satellites used in positioning in Taipei FIR (3 RSs).

For the WADGPS system with three RSs in Taipei FIR, Figure 21 shows the positioning error distributions in both east and north directions, and Figure 22 shows the vertical positioning errors distribution. As for the integrity of this WADGPS implementation, the HPL values effectively bound the horizontal positioning errors, as shown in Figure 23. As depicted in Figure 24, the LNAV service availability is 99.977% over the five-day period for the developed WADGPS system with three RSs. As shown in Figure 24, there is no data sample located in the red region (*i.e.,* the hazardously misleading information (HMI) region) of the figure, in other words, the horizontal protection level (HPL) calculated by this WADGPS architecture successfully bound the horizontal positioning error. As a result, the integrity (defined in Section 2) of this WADGPS implementation is ensured. The number of satellites used in the solutions is shown in Figure 25.

**Figure 26.**
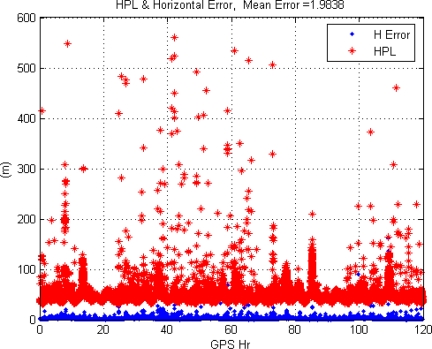
The HPL and horizontal position error in Taipei FIR (4 RSs).

**Figure 27.**
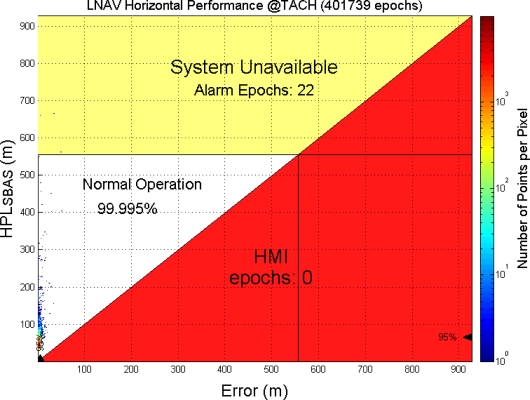
The LNAV (NPA) performance of the developed WADGPS with 4 RSs in Taipei FIR.

To achieve possible improvement of the system performance, this WADGPS implementation adds one more e-GPS observation station (Station number 4 in Table 4) to be the fourth RSs. Figure 26 shows that the HPL values also bound the horizontal positioning errors successfully. Figure 27 shows the Stanford Chart of the WADGPS system with four RSs. In comparison to Figure 24, the total number of the epochs is increased from 380,142 epochs to 401,739 epochs (*i.e.*, 21,597 more epochs), and the LNAV service availability is improved from 99.977% to 99.995%. Additionally, system unavailable epochs is reduced from 86 to 22. Tables 5 and 6 summarize the comparison of the positioning performance. The results show that the WADGPS system with four RSs performs slightly better than the WADGPS system with three RSs.

## 6. Conclusions

This paper implemented a Wide Area Differential Global Positioning System (WADGPS) system in Taipei Flight Information Region. The National Satellite Test Bed (NSTB) Reference Stations (RSs) were first used as the WADGPS RSs to validate the implementation. As shown in the three days validation results, the WADGPS system can provide enhanced GPS positioning services with full integrity required by the Lateral NAVigation (LNAV) service for civil aviation. This paper then used the e-GPS observation stations operated by Taiwan Ministry of Interior (MOI) as the WADGPS RSs in Taipei FIR. Two kinds of WADGPS RSs constellations were utilized in this work, and one used three RSs and the other used four RSs. Five-day data were used to analyze both WADGPS implementations. The results showed that the WADGPS system with four RSs performed slightly better than that with three RSs. Importantly, in Taipei FIR, both WADGPS implementations can successfully provide LNAV service with integrity required by civil aviation.

## Figures and Tables

**Table 1.** Required Navigation Performance (RNP) [6,7].

**Phase of Flight**	**Accuracy (95% error)**	**Integrity**		**Alert Limit (H: Horizontal V: Vertical)**	**Continuity**	**Availability**
		**Time to Alarm**	**Pr(HMI)**			

En route (continental)	H: 740 m V: NA	15 s	1 × 10^−7^ /h	H: 3,704 m V: NA	1 × 10^−5^/hr	0.99 to 0.99999
Terminal	H: 220 m V: NA	15 s	1 × 10^−7^ /h	H: 1,852 m V: NA	1 × 10^−5^/h	0.99 to 0.99999
LNAV(NPA)	H: 220 m V: NA	10 s	1 × 10^−7^ /h	H: 556 m V: NA	1 × 10^−5^/h	0.99 to 0.99999
LNAV/VNAV	H: 220 m V: 20 m	10 s	2 × 10^−7^ /approach	H: 556 m V: 50 m	5.5 × 10^−5^ /approach	0.99 to 0.999
LPV	H: 16 m V: 20 m	6 s	2 × 10^−7^ /approach	H: 40 m V: 50 m	5.5 × 10^−5^ /approach	0.99 to 0.99999

**Table 2.** Mean of the positioning error (NSTB).

**Mean**	**Stand alone GPS**	**WADGPS**

**East (m)**	−0.506	0.090
**North (m)**	0.436	−0.085
**Vertical (m)**	15.707	−7.968

**Table 3.** Accuracy of the positioning performance (NSTB).

**95% error bound (two-sigma)**	**Stand alone GPS**	**WADGPS**

**Horizontal (m)**	7.10	3.55
**Vertical (m)**	16.10	8.10

**Table 4.** The e-GPS observation stations’ names and locations.

**Station No.**	**Station name**	**Latitude**	**Longitude**

1	Longdong	25° 5′50″N	121° 55′5″E
2	Shoufeng	23° 52′12″N	121° 36′53″E
3	Fugang	22° 47′26″N	121° 12′32″E
4	Kaohsiung	22° 37′52″N	120° 17′18″E
User	Taichung	24° 17′27″N	120° 32′6″E

**Table 5.** Mean of positioning error (Taipei FIR).

**Mean error**	**Stand alone GPS**	**WADGPS with 3 RSs**	**WADGPS with 4 RSs**

**East (m)**	0.182	0.220	0.169
**North (m)**	1.785	1.362	1.498
**Up (m)**	13.400	−2.431	−2.440

**Table 6.** Accuracy of positioning performance (Taipei FIR).

**95% error bound (two-sigma)**	**Stand alone GPS**	**WADGPS with 3 RSs**	**WADGPS with 4 RSs**

**Horizontal (m)**	10.970	5.587	4.0893
**Vertical (m)**	19.360	11.248	11.485
